# “Cellularity as a predictive tool for mesenchymal stem cell concentration in bone marrow concentrates: Implications for regenerative medicine”

**DOI:** 10.1016/j.bonr.2024.101820

**Published:** 2024-11-26

**Authors:** Klaus Werner Labarre, Peter Ansgar Grathwol, Gerald Zimmermann

**Affiliations:** Department of Trauma Surgery, Theresienkrankenhaus, Bassermannstraße 1, 68165 Mannheim, Germany

**Keywords:** Mesenchymal stem cells, Bone marrow concentrate, Cellularity, Regenerative medicine, Flow cytometry, CD271^+^ cells

## Abstract

**Background:**

Mesenchymal stem cells (MSCs) derived from bone marrow play an increasingly important role in regenerative medicine due to their capacity to promote tissue regeneration in various clinical contexts. Applications include the treatment of osteoarthritis, bone regeneration post-injury, and the management of conditions such as Crohn's disease, alopecia, and nervous system reconstruction. Accurate quantification of MSCs within Bone Marrow Concentrates (BMCs) is essential for ensuring the quality and efficacy of these cell therapy products in clinical settings.

**Objective:**

This study aims to quantify the population of CD271^+^ and CD45^−^ cells in BMCs prepared using the method we have selected and to provide a basis for comparing these results with other BMC products. Additionally, we seek to determine whether the total cell count in BMCs can serve as a reliable indicator of MSC numbers and if cellularity (the number of cells per ml) can predict a higher percentage of MSCs within the population.

**Methods:**

Bone Marrow Aspirates (BMA) were collected from 41 patients undergoing knee or hip arthroplasty. Aspirates were processed using density gradient centrifugation and positive selection of CD271^+^ cells. Flow cytometry was applied to analyze cell subsets, and cell counts were determined with a NucleoCounter. The relationships between BMA cellularity (total cells per ml), MSC concentration (MSC count per ml), and MSC percentage (the proportion of MSCs within the total cell population) were assessed.

**Results:**

The mean percentage of CD271^+^ CD45^−^ cells in bone marrow samples was 0.03 % (SD 0.03 %). Cellularity varied significantly among samples, with a mean of 6 million cells/ml (SD 8.7 million cells/ml). A strong correlation was observed between BMC cellularity and MSC concentration (*p* < 0.05), although no correlation was found between cellularity and the MSC percentage.

**Conclusion:**

Despite high variability in cellularity, the concentration of MSCs correlated strongly with BMC cellularity, suggesting that total cell counts can be used to estimate MSC numbers in BMCs. However, cellularity is not an indicator of a particularly high MSC content. This study supports the use of cell counts as a measure for estimating MSC concentration in BMCs. Future research should focus on establishing direct comparisons with other BMC products and exploring factors influencing cellularity and MSC percentages to enhance BMC quality for clinical applications.

## 1 Introduction

Cell therapy employs mesenchymal stem cells (MSCs) across various clinical applications to promote tissue regeneration. These applications include the treatment of osteoarthritis, bone regeneration post-injury, and the management of conditions such as Crohn's disease, alopecia, and nervous system reconstruction. Additionally, MSCs are utilized in myocardium restoration, liver regeneration, and corneal reconstruction (Han et al., 2019; Dryden, 2009; Stevens et al., 2018).

Bone marrow-derived multipotent stromal cells, are highly proliferative and capable of generating skeletal tissues, including bone and cartilage (Bianco et al., 2010; Deschaseaux et al., 2010).

MSCs can be used in a one-step procedure as minimally manipulated MSCs or after in vitro expansion. While in vitro expansion allows for a more homogeneous cell population that meets standard MSC identification criteria, it is also associated with increased costs, time requirements, and potential issues such as senescent cell accumulation, telomere erosion, and phenotype changes (Lv et al., 2014; Jones et al., 2010; Halfon et al., 2011; Wagner et al., 2008; Baxter et al., 2004). Moreover, in vitro-expanded cells may lose their ability to migrate to target tissues through chemokine receptor interactions, known as the “homing effect” (Khaldoyanidi, 2008; Sohni and Verfaillie, 2013). Using minimally manipulated MSCs from bone marrow or Stromal vascular fraction (SVF) from adipose tissue may help overcome these disadvantages (Di Matteo et al., 2019).

SVF and bone marrow concentrate (BMC) are the two most important therapies involving minimally manipulated cells that can be applied in a one-step procedure, contrasting with MSC therapies that require in vitro expansion. Current studies have demonstrated promising results for both SVF and BMC in the treatment of knee osteoarthritis (OA). El-Kadiry et al., (2022) describes in their study that BMC provided superior outcomes in terms of pain relief and functional improvement over a one-year follow-up period. SVF has shown significant pain reduction and functional improvements, with evidence of cartilage regeneration in some cases, making it a potentially valuable approach for managing OA (Boada-Pladellorens et al., 2022). Similarly, BMC has been effective in improving pain, function, and quality of life, often demonstrating long-term benefits up to two years post-treatment (Centeno et al., 2018; Goncars et al., 2018). Burnham et al., (2021) reported significant improvements in pain, disability, and quality of life after BMC injections in patients with refractory OA, with a low complication rate. Bolia et al., (2021) suggest that SVF may offer superior pain relief compared to BMC, though more standardized methods and larger trials are necessary to confirm these findings. Goncars et al., (2018) also demonstrated that bone marrow-derived mononuclear cells (BM MNCs) could partially reduce OA symptoms and degeneration in some cases. Klingenberg et al. (2024) found that combining SVF with autologous conditioned plasma significantly improved clinical symptoms in patients with advanced knee OA. Ren et al. (2023) provided evidence for cartilage regeneration using SVF, highlighting its potential for structural improvement in OA treatment. Prizov et al., 2024) observed that combining high tibial osteotomy (HTO) with SVF post-treatment led to enhanced outcomes in bone architecture. Agarwal et al., (2021) also confirmed that SVF and adipose-derived mesenchymal stem cells (AMSCs) reduce pain and improve function, supporting their potential for enhancing mobility in an aging population. Overall, SVF and BMC represent promising minimally invasive treatment options for OA, with distinct benefits that warrant further research to establish their optimal use. However, many different methods are used to produce SVF and BMC, with limited valid data on cell counts or the subpopulations present in the products. Much foundational research is needed to improve comparability in the future.

In the identification and characterization of mesenchymal stem cells (MSCs), a variety of surface markers are utilized to define their properties and differentiation potential. Among these, CD73, CD90, and CD105 are commonly employed and are essential for confirming the MSC phenotype, in accordance with the guidelines established by the International Society for Cellular Therapy (ISCT). (Maldonado et al., 2024; Zhao et al., 2019). CD73 is involved in the regulation of immune responses, while CD90 (Thy-1) is associated with cell adhesion and signaling (Zhao et al., 2019). CD105, also known as endoglin, is crucial for angiogenesis and is often used to assess the differentiation potential of MSCs (Maldonado et al., 2024). Other markers such as CD44 and CD29 are indicative of the cells' ability to adhere to extracellular matrix components (Zhao et al., 2019). The expression of CD146 (MCAM) has been linked to the isolation of potent MSCs with enhanced chondrogenic differentiation capabilities (Kanawa et al., 2019). Importantly, the absence of hematopoietic markers like CD45, CD34, and CD14 further confirms the mesenchymal phenotype of these cells (Zhao et al., 2019; Pham et al., 2019).

Several reports (Quirici et al., 2002; Mabuchi et al., 2013; Harichandan and Bühring, 2011) indicate that among these markers CD271 expression is linked to the most homogeneous subset of MSCs, capable of differentiating into adipogenic, osteogenic, and chondrogenic lineages, as well as producing significantly higher cytokine levels compared to plastic adherence-mesenchymal stromal cells (PA-MSCs) (Jones et al., 2010; Calabrese et al., 2015; Tormin et al., 2011). CD271, originally described as a nerve growth factor receptor (NGFR) and also known as LNGFR (low-affinity nerve growth factor receptor), belongs to both the low-affinity neurotrophin receptor family and the tumor necrosis factor receptor superfamily (Johnson et al., 1986; Thomson et al., 1988). It interacts with neurotrophins such as brain-derived neurotrophic factor (BDNF), neurotrophin-3, and neurotrophin-4, as well as with pro-neurotrophins (proNTs) like proNGF and proBDNF (Johnson et al., 1986; Thomson et al., 1988; Hempstead, 2014). This antigen is found in various cell types, including neurons, Schwann cells, mesenchymal stem cells, follicular dendritic cells, and melanocytes, among others (Thomson et al., 1988; Maria et al., 2013). Within the nervous system, CD271 is believed to play a role in neural cell development, survival, and differentiation (Thomson et al., 1988; Hempstead, 2014; Casaccia-Bonnefil et al., 1999; Yan et al., 1991). Additionally, CD271+ MSCs have demonstrated higher proliferation potential compared to the entire PA-MSC population (Maria et al., 2013). However, CD271 has also been identified as a marker of tumor-initiating cells, potentially influencing cell survival and proliferation (Civenni et al., 2011; Imai et al., 2013; Murillo-Sauca et al., 2014). The observed capacity of CD271+ MSCs to effectively differentiate into cartilage tissue (Boada-Pladellorens et al., 2022; Mabuchi et al., 2013; Harichandan and Bühring, 2011) was a key factor in our decision to focus on this marker, as cartilage regeneration represents one of the most important applications in orthopedics.

To qualify cell therapy products as “advanced medical products,” it is crucial to verify the quality of the specific product. One method to assess product quality is by quantifying MSCs. CD271^+^ CD45^−^ cells, which are the population of interest in minimally manipulated Bone Marrow Aspirate (BMA), remain a rare cell population. Literature reports indicate that the percentage of CD271^+^ cells in BMA ranges from 0.001 % to 0.1 %, with significant variability between samples (Alvarez-Viejo et al., 2013; Cuthbert et al., 2012).

Measuring such rare cell populations can be performed using flow cytometry, which is time-consuming and requires a large volume of bone marrow. In the clinical setting, it is crucial to inject the cell product promptly after collection to minimize the risks of contamination and apoptosis. Furthermore, such quality assessments should be straightforward to perform clinically. There is a consensus among surgeons that achieving a high cell number and viability is essential. The cellularity of bone marrow-derived mononuclear cells per volume and their viability can be quickly and easily determined using a NucleoCounter. However, more detailed analyses of the cell product are necessary to determine the significance of these measurements.

This study aims to collect quantitative data on the cellularity and composition of MSCs to support further investigations and clinical applications. Given the expected high variation in cellularity and MSC percentages, it is essential to evaluate cellularity as a measure for estimating MSC proportions. These data are crucial for developing and standardizing Bone Marrow Concentrate (BMC) products. Although various medical devices for BMC production have emerged, data on the composition and quality of these therapeutics are limited and often controversial. Specifically, there is limited information on the proportion of CD271^+^ cells in BMCs for clinical application due to the difficulty of detecting such rare populations. This study seeks to quantify CD271^+^ CD45^−^ cells in BMCs produced using our selected method and establish a basis for comparison with other products. Additionally, we aim to determine if total cell count can reliably indicate MSC numbers for simplified future measurements and whether high cellularity samples indicate a higher MSC proportion. By achieving these objectives, we aim to enhance the reliability and efficacy of BMCs in clinical settings.

## 2 Materials and methods

### 2.1 Harvest of BMA

**Fig. 1:**
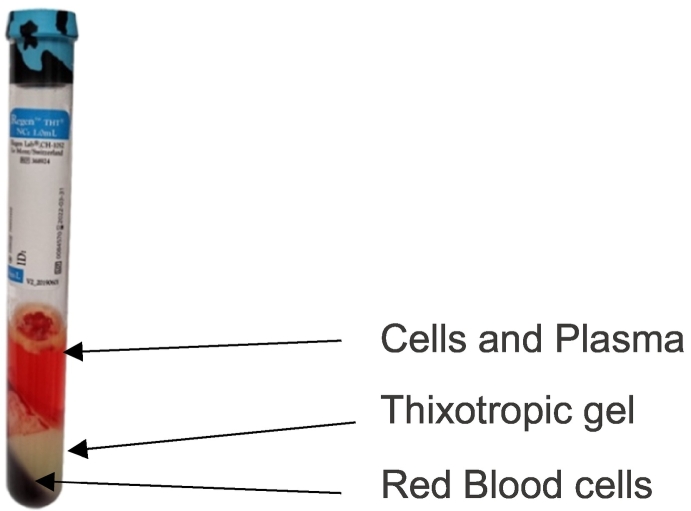
Tube with Bone Marrow Aspirate after centrifugation.

BMA was obtained from the proximal or distal femur or proximal tibia during elective knee or hip arthroplasty. In patients who underwent knee arthroplasty, samples from the distal femur and proximal tibia were analyzed separately. During the operation, before the respective bone was opened, the corresponding harvesting site on the bone was punctured at two different locations using a Jamshidi needle, and 6 ml of bone marrow was aspirated two times. After aspirating 3 ml, the position of the needle was altered by pushing deeper into the bone or pulling backward. The aspiration syringes and the Jamshidi needle were rinsed with a Heparin/NaCL solution(10.000 Units/ml) beforehand. After collection, each 6 ml aspirate was transferred into a separate RegenExtracell tube (RegenLab, Brooklyn, NY, USA). The RegenExtracell tube contains a thixotropic gel that acts as a density gradient for the depletion of red blood cells, with an anticoagulant on top to prevent blood clotting. The tubes were then inverted 20 times and centrifuged for 9 min at room temperature according to the manufacturer's instructions. After density gradient centrifugation, the majority of the red blood cells remain below the gel layer while the rest of the cells and plasma can be seen on top of it. The tubes were again inverted several times to mix the supernatant well (Fig. 1). The latter was then placed in a 15 ml Falcon tube for further processing.

### 2.2 Erythrocyte lysis and positive selection

The cells were centrifuged, and the supernatant was discarded except for one milliliter. Red Blood Cell (RBC) lysis was performed by adding 10 ml of RBC Lysis Buffer (Invitrogen by Thermo Fisher Scientific, Waltham, MA, USA), with an incubation time of 5 min at room temperature. The sample was then centrifuged twice at 300 ×*g* for 10 min, and the cell pellet was collected in buffer solution? (consisting of phosphate-buffered saline (PBS), pH 7.2, 0.5 % bovine serum albumin (BSA), and 2 mM ethylenediaminetetraacetic acid (EDTA)).

In the next step, CD271^+^ cells were isolated from the remaining cell pellet using the CD271 MicroBead Kit (Miltenyi Biotec, Bergisch Gladbach, Germany) according to the manufacturer's protocol. Specifically, the cell suspension was resuspended in 60 μl of buffer (PBS, pH 7.2, 0.5 % BSA, and 2 mM EDTA). Then, 20 μl of FcR Blocking Reagent and 20 μl of CD271 MicroBeads were added. The mixture was incubated for 15 min at 2–8 °C. The cells were washed by adding 4,5 ml of buffer and centrifuged at 300 ×*g* for 10 min. The cell pellet was resuspended in 500 μl of buffer. The cell suspension was then loaded onto an MS Column (Miltenyi Biotec, Bergisch Gladbach, Germany), a magnetic separation column used to retain magnetically labeled cells, which was placed in a MiniMACS Separator (Miltenyi Biotec, Bergisch Gladbach, Germany). The column was washed three times with 500 μl of buffer, and the magnetically labeled CD271^+^ cells were eluted with 1 ml of buffer by firmly pushing the plunger into the column. Finally, 1 ml of cell solution containing the positively selected cells and approximately 2,5 ml of flow-through solution containing the non-selected cells were collected.

### 2.3 Cell count

Cell counts were determined using a Nucleo-Counter NC-200® cell counter (ChemoMetec A/S, Allerod, DK, Denmark). For the first cellcount directly after the density gradient centrifugation(n1), 20 μl of the untreated sample was taken from the Falcon tube and mixed with 180 μl of Solution 17 (ChemoMetec A/S, Allerod, DK, Denmark) to lyse the remaining erythrocytes. This was followed by incubation for 10 min, and then 60 μl of this sample was loaded into a Via-1 cassette, which stains nucleoli of dead cells with 4′,6-diamidine-2-phenylindole (DAPI), this number measerued the number of cell per milliliter of BMC intended for the clinical use. The NucleoCounter determined the number of viable cells and the total number of nucleated cells per milliliter (n1live; n1). A second measurement (n1liven2) was taken directly before the positive selection from 500 μl containg all cell of the original sample. After the positive selection, both the number of selected cells (1 ml) and the number of cells in the flowthrough were determined. (n3live, nflive,n3, nf,).

### 2.4 Staining of the surface proteins and fluorescence measurement

Multiparameter flow cytometry was used to analyze the CD271 positive selected cells. The cells were distributed onto 4 labeled 1.5 ml Eppendorf tubes and centrifuged to perform antibody staining. The cell pellet was resuspended in 25 μl PBS buffer and then stained with 0.5 μl of the respective antibodies (Miltenyi Biotec, Bergisch Gladbach, Germany as detailed in Table 1). One sample was left unstained. The primary sample was stained with CD45 FITC and CD45 APC; in two samples, one antibody was replaced by an isotype control. After 10 min of incubation at 2–8 °C in the dark, the cells were washed twice with 1 ml of PBS Buffer and resuspended in 200 μl of PBS Buffer. Each sample was then incubated with 5 μl of 7AAD (Invitrogen eBioscience) for 5–15 min at room temperature. Flow cytometry was performed using a Guava easyCyte™ 8HT (Luminex Corp. Austin, Texas, United States). The device was checked for accuracy before each measurement using the Guava easyCheck™ kit (Luminex Corp. Austin, Texas, United States). The gain controls of the instrument were adjusted at the beginning of the experiment series using single stained Controls for CD271 and CD45. Isotype controls confirmed the gating position for all cell populations studied and excluded unspecific binding. Each sample was analyzed for 7 min.

**Table 1:** Antibody conjugates used for flow cytometry.

Reagent	Company	Clone
CD271 (LNGFR) Antibody, anti-human/mouse, APC, REAfinity™	Miltenyi Biotec	REA648
CD45 Antibody, anti-human, FITC, REAfinity™	Miltenyi Biotec	REA747
REA Control Antibody (S), human IgG1, APC, REAfinity™	Miltenyi Biotec	REA293
REA Control Antibody (S), human IgG1, FITC, REAfinity™	Miltenyi Biotec	REA293

Due to the lower event rate of the Guava easyCyte™ 8HT flow cytometer, which necessitated a setting of approximately 30 events per second for optimal results, we were unable to achieve the high event rates used in other studies, such as Cuthbert et al. (2012), who set the event rate to 2000 events per second. As a result, to reliably detect the rare CD271^+^ cell population, we performed a positive selection step using magnetic cell separation. This step was crucial to ensure accurate detection of these rare cells but likely contributed to additional cell loss during processing.

### 2.5 Analysis of flow cytometry data for enumeration assay

Flow cytometry data were analyzed using Guava® InCyte™ software version 4.0. (Luminex Corp., Austin, Texas, United States). Cell debris was excluded based on forward and side scatter, and the uptake of 7AAD identified dead/dying cells. Cell subsets were identified as CD271^+^ cells (CD45^−^ CD271^+^) and CD45+ cells. The population size was expressed as a percentage of the MSCs of the fraction after positive selection (n3). In order to calculate the percentage of MSCs in the original sample (n1) intended for clinical use, the following formula was applied:

$$\text{Pcells}=\frac{\left(\frac{\mathrm{pcells}*n3}{100}\right)*100}{n2}$$

Equation 1: Pcells is the proportion of the specific cell population in all vital mononucleated cells of the original sample intended for clinical use. Pcells is the percentage of the specific cell population in the cell fraction after positive selection(n3), n3 is the total number cells after positive selection, n2 is the total number of cells measured before positive selection containing all cells from the orginal sample.

### 2.6 Statistics

The Shapiro-Wilk normality test and QQ plots were used to assess the normality of data distributions and to determine appropriate correlation and significance tests. Results showed that none of the cell counts in Table 3, Table 4, Table 5 including total live cell counts, CD271^+^ cell counts, CD271^+^CD45- counts, and CD271^+^CD45^+^ counts followed a normal distribution. Consequently, these values are presented as medians and interquartile ranges (IQR) in Table 3, Table 4, Table 5. Age, however, was normally distributed (W = 0.9595, *p* = 0.1516), so it is reported as a mean and range (minimum-maximum). All correlations in Fig. 3 were analyzed using non-parametric methods, specifically the Spearman rank correlation test, to account for the non-normal distributions. Statistical outliers were defined as values 1.5 times the interquartile range above the 75th percentile or below the 25th percentile. These outliers were reviewed for potential data acquisition errors. Extreme outliers were removed from Fig. 3 to improve graphical clarity without affecting the statistical significance of the results. Other outliers, deemed logically explainable, were retained in the analysis. Both Spearman's rank correlation and Pearson correlation tests were used where applicable to measure statistical dependence and to generate 95 % confidence intervals for predicted values. Statistical significance was defined as *p* < 0.05. All statistical analyses and graphs were produced using RStudio (RStudio Team, 2021).

### 2.7 Gating strategy and control of nonspecific binding

**Fig. 2:**
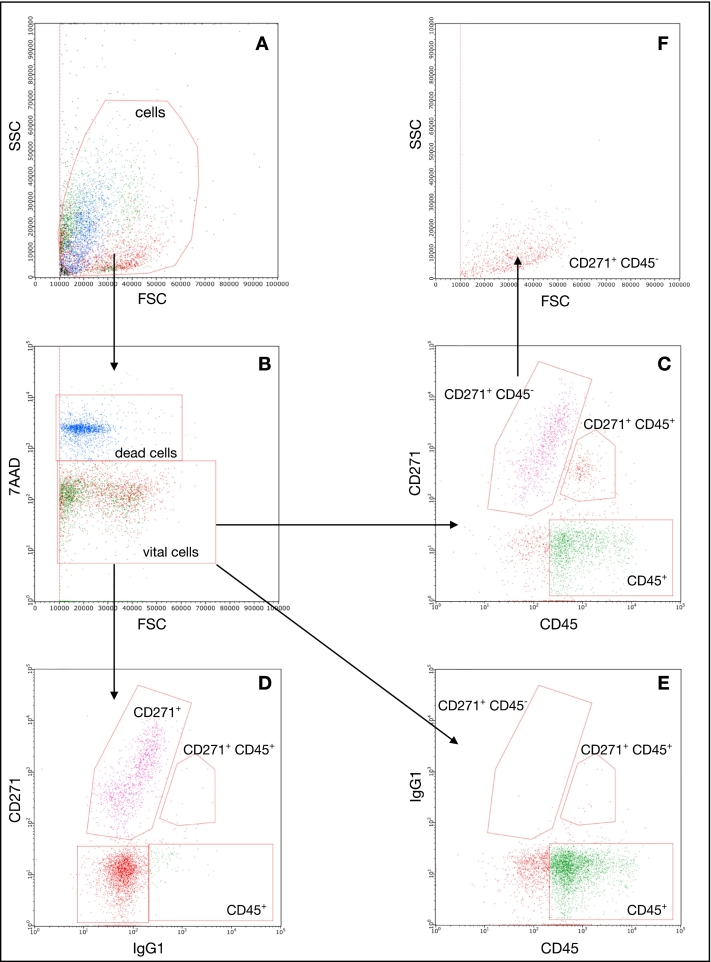
Representative example: Gating strategy for enumeration of CD 271 ^+^ cells. (A) Cell debris is based on forward and sideward scatter. (B) Dead cells are gated out based on their uptake of 7AAD. (C) CD271^+^ cells are enumerated on CD45/CD721 dot-plot. (D, E) Minimal non-specific binging of CD27 ^+^/CD45 ^+^cells is observed when IgG isotype is used instead of CD271/CD45 antibody. (F) The CD 271^+^ CD45 ^−^ cells population is gated into the FSC/SSC plot.

In our experiment, we employed a scatter threshold and a polygonal gate to exclude cell debris (Fig. 2A). Viability was assessed using 7-AAD, allowing us to exclude dead or dying cells based on 7-AAD positivity. IgG isotype control groups were utilized to ensure the absence of nonspecific binding and to adjust the placement of the statistical markers (Fig. 2C, D). CD271^+^ cells were readily identified and quantified using individual polygonal statistical markers on a CD45/CD271 dot-plot (Fig. 2E).

## 3 Results

**Fig. 3:**
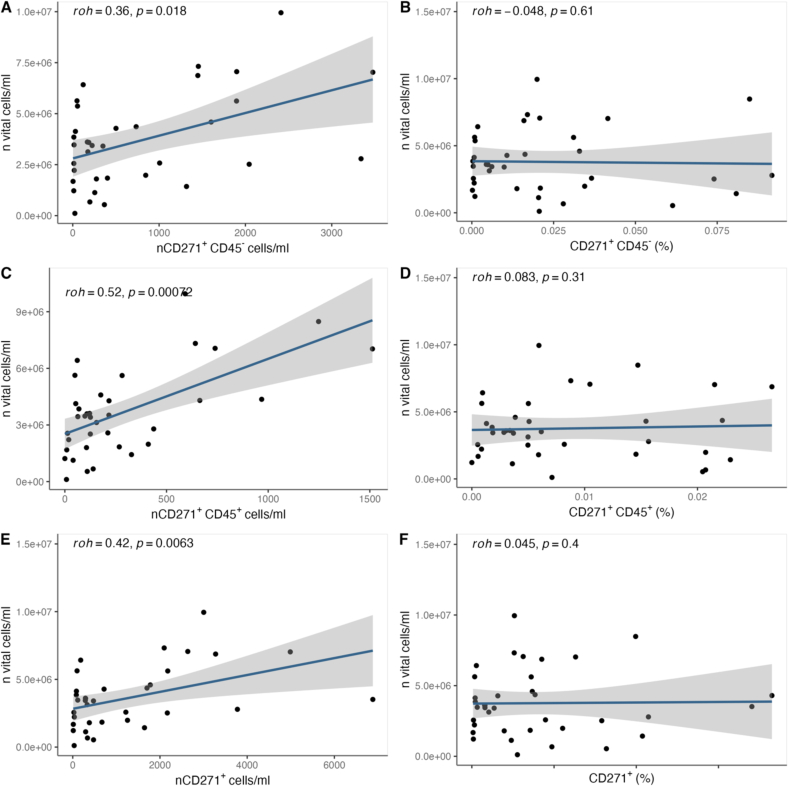
Correlation between overall cellularity and total MSC concentration as well as the percentage of BM-MNCs: A/C/E show a strong positive correlation between the cellularity and the concentration of MSCs. There was no significant correlation between the cellularity and the percentage of MSCs (B/D/F).

The targeted CD271^+^ CD45^−^ MSCs could be detected after positive selection to expected levels. Besides, a population of CD271^+^ CD45^+^ cells was also shown, as previously described by Cuthbert et. Al. (Cuthbert et al., 2012). There were significant variations in both the general cellularity and the percentage of MSCs of the BMC (tabel3). Cellularity correlated strongly with the concentration of CD271^+^ as well as CD271^+^CD45^−^, CD271^+^CD45^+^ cells (*p* < 0.05) (Fig. 3 A,C,E). However, no correlation could be found between cellularity and the percentage of these MSCs (Fig. 3 B,D,F).

### 3.1 Patient demographics and characteristics by collection site

There were no significant differences between samples from different collection sites with respect to patient demographic characteristics (Table 2).

**Table 2:** Comparison of patient demographic characteristics between samples collected from different anatomical sites (distal femur, proximal femur, proximal tibia). The table shows the mean age (years), gender distribution, walking distance (wd) categorized as <500 m or ≥ 500 m, and body mass index (BMI) for each collection site. *P*-values indicate the statistical significance of differences between the groups, calculated using the Kruskal-Wallis rank sum test and Fisher's exact test..

Variable	N	Distal femur, N = 16^1^	Proximal femur, N = 10^1^	Proximal tibia, N = 15^1^
Age	41	71 (55, 86)	71 (57, 82)	69 (55, 82)
Gender	41			
Female		9 / 16 (56 %)	5 / 10 (50 %)	9 / 15 (60 %)
Male		7 / 16 (44 %)	5 / 10 (50 %)	6 / 15 (40 %)

1 Mean (Range); n / N (%).

### 3.2 Cell numbers and percentages of MSCs in bone marrow concentrate

The targeted CD271^+^ CD45^−^ MSCs could be detected after positive selection to expected levels. Besides, a population of CD271^+^ CD45^+^ cells was also shown as previously described by Cuthbert et al., (2012). There were large variations in both the general cellularity and the percentage of MCPs of the BMC (Table 3).

**Table 3:** Cell concentration and percentages of mesenchymal stromal cells (MSCs) in bone marrow concentrate (BMC), including vital and total nucleated cells per milliliter, CD271^+^CD45^−^ and CD271^+^CD45^+^ populations, and total CD271^+^ cells. Values are presented as means with standart deviation(SD).

Cell counts and population percentages	Median [IQR]
n vital nucleated cells/ml BMC	3.59e+06 [2.22e+06, 5.63e+06]
n total number of nucleated cells/ml BMC	3.82e+06 [2.45e+06, 6.1e+06]
CD271^+^ CD45^−^ population (%)	0.014 [0.004, 0.036]
CD271^+^ CD45^+^ population (%)	0.004 [0.002, 0.012]
CD271^+^ (%)	0.021 [0.007, 0.047]
Total number of CD271^+^ CD45^−^ cells/ml BMC	2.8e+02 [1.12e+02, 1.36e+03]
Total number of CD271^+^ CD45^+^ cells/ml BMC	1.05e+02 [5.12e+01, 4.29e+02]
Total number of CD271^+^ cells/ml BMC	4.23e+02 [1.88e+02, 2.26e+03]

### 3.3 Comparison of cell counts and cell populations by bone marrow aspiration sites

The comparison of cell counts and specific cell populations across bone marrow aspiration sites—distal femur (df), proximal femur (pf), and proximal tibia (pt)—revealed notable differences (see Table 4). For *vital nucleated cells/ml*, the cell concentration was significantly higher in the proximal femur (pf) compared to the distal femur (df), as well as in the pf compared to the proximal tibia (pt), with adjusted *p*-values of 0.0018 and 0.0023, respectively.

**Table 4:** This table presents pairwise comparisons of cell concentrations and proportions of specific cell populations in bone marrow aspirates collected from three anatomical sites: distal femur (df), proximal femur (pf), and proximal tibia (pt). Concentrations (cells/ml) reflect the density of each cell population within the aspirate, while percentages (%) represent the proportion of each population within the total cell count. Statistically significant differences were determined using the Dunn-Test with Bonferroni adjustment, with significance levels denoted by *** *p* < 0.001, ** *p* < 0.01, and * *p* < 0.05.

Variable	Aspiration site	*Z*-score	Adjusted p-value	Significance
Vital nucleated cells/ml	df - pf	−3.4324	0.0018	**
Vital nucleated cells/ml	df - pt	−0.0203	1.0000	
Vital nucleated cells/ml	pf - pt	3.3656	0.0023	**
CD271 ^+^ CD45 ^−^ (%)	df - pf	−2.1897	0.0856	
CD271 ^+^ CD45 ^−^ (%)	df - pt	0.4321	1.0000	
CD271 ^+^ CD45 ^−^ (%)	pf - pt	2.5601	0.0314	*
CD271 ^+^ CD45 ^−^ (cells/ml)	df - pf	−3.2498	0.0035	**
CD271 ^+^ CD45 ^−^ (cells/ml)	df - pt	0.0628	1.0000	
CD271 ^+^ CD45 ^−^ (cells/ml)	pf - pt	3.2626	0.0033	**
CD271 ^+^ CD45 ^+^ (%)	df - pf	−2.4287	0.0455	*
CD271 ^+^ CD45 ^+^ (%)	df - pt	0.7543	1.0000	
CD271 ^+^ CD45 ^+^ (%)	pf - pt	3.0947	0.0059	**
CD271 ^+^ CD45 ^+^ cells/ml	df - pf	−3.3150	0.0027	**
CD271 ^+^ CD45 ^+^ cells/ml	df - pt	0.3961	1.0000	
CD271 ^+^ CD45 ^+^ cells/ml	pf - pt	3.6362	0.0008	***
CD271 ^+^ (%)	df - pf	−2.2723	0.0692	
CD271 ^+^ (%)	df - pt	0.4626	1.0000	
CD271 ^+^ (%)	pf - pt	2.6698	0.0228	*
CD271 ^+^ cells/ml	df - pf	−3.3280	0.0026	**
CD271 ^+^ cells/ml	df - pt	0.0499	1.0000	
CD271 ^+^ cells/ml	pf - pt	3.3278	0.0026	**

Dunn-Test with Bonferroni adjustment was used for pairwise comparisons.

Significance codes: *** p < 0.001, ** p < 0.01, * p < 0.05.

In terms of CD271^+^ CD45- (%), representing the proportion of CD271^+^ CD45 ^−^ cells within the total cell population, there was a significantly higher percentage in the proximal femur (pf) compared to the proximal tibia (pt) (*p* = 0.0314). Similarly, CD271 ^+^ CD45 ^−^ (cells/ml), the concentration of these cells in the bone marrow aspirate, was significantly higher in the pf site compared to both the distal femur (df) (*p* = 0.0035) and the proximal tibia (pt) (*p* = 0.0033).

CD271 ^+^ CD45 ^+^ (%)—reflecting the proportion of CD271 ^+^ CD45 ^+^ cells within the total cell population—was also significantly higher in the pf site compared to the df (*p* = 0.0455) and pt. (*p* = 0.0059). For CD271^+^ CD45^+^ (cells/ml), the concentration was again higher in pf compared to df (*p* = 0.0027) and pt. (*p* = 0.0008), with the latter comparison indicating a particularly strong statistical significance (***).

The percentage of CD271^+^ cells within the total population (CD271^+^ (%)) was significantly greater in the pf than in the pt. site (*p* = 0.0228). Lastly, for the concentration of CD271^+^ cells/ml, the pf site demonstrated a higher concentration than both the df and pt. sites, with significant *p*-values of 0.0026 for both comparisons.

The analysis showed that both the overall cell count (vital nucleated cells/ml) and the concentration of CD271^+^ cells were higher in the proximal femur (pf) site compared to the distal femur (df) and proximal tibia (pt). The pf site also exhibited a higher percentage and concentration of CD271^+^ CD45- cells, as well as an increased proportion and concentration of CD271^+^ CD45^+^ cells relative to df and pt.

These results in Table 4 indicate that the proximal femur yields a higher concentration of both total nucleated cells and specific CD271^+^ cell populations (CD45 ^−^ and CD45 ^+^) compared to the other aspiration sites.

### 3.4 Cell loss during sample preparation

A detailed analysis of the total and live cell counts at three different time points (n1, n2, and n3) was conducted, as well as the cell loss and percentage of cells lost between these time points. The following steps were taken to calculate these values.

First, the total and live cell counts were measured at three different points in time: n1, n2, and n3. For each time point, the absolute cell loss was calculated by subtracting the total cell count at the later time point from the total cell count at the earlier time point. This was done for both total and live cells. Additionally, the percentage of cells lost was calculated by dividing the cell loss by the total cell count at the earlier time point and then multiplying by 100.

The results are summarized in Table 5. The table presents the mean and standard deviation for each parameter measured. Specifically, the table includes the total cells and live cells at each time point, the absolute cell loss between time points, and the percentage of cells lost between time points. This analysis provides insight into the dynamics of cell loss over time.

**Table 5:** Presents a summary of the cell counts and cell losses at three different time points (n1, n2, and n3). The table includes both absolute and percentage losses for total and live cells. The data is presented as mean values with standard deviations in parentheses.

Measurement	Median [IQR]
Total Cells at n1	3.44e+07 [2.19e+07, 4.99e+07]
Total Cells at n2	1.94e+07 [9.95e+06, 3.12e+07]
Total Cells at n3	1.68e+07 [8.34e+06, 3.04e+07]
Live Cells at n1	3.27e+07 [1.98e+07, 4.49e+07]
Live Cells at n2	1.9e+07 [8.65e+06, 2.86e+07]
Live Cells at n3	1.62e+07 [8.17e+06, 2.88e+07]
Total Cells Lost between n1 and n2	1.01e+07 [2.45e+06, 2.13e+07]
Total Cells Lost between n2 and n3	2.01e+06 [−1.96e+06, 5.59e+06]
Total Cells Lost between n1 and n3	1.21e+07 [5.21e+06, 1.92e+07]
Live Cells Lost between n1 and n2	8.62e+06 [4.38e+06, 1.61e+07]
Live Cells Lost between n2 and n3	2.23e+06 [−2.31e+06, 4.85e+06]
Live Cells Lost between n1 and n3	1.09e+07 [3.34e+06, 1.62e+07]
Total Cells Lost between n1 and n2 (%)	37.5 [18.1, 57.2]
Total Cells Lost between n2 and n3 (%)	16.2 [−9.9, 34.7]
Total Cells Lost between n1 and n3 (%)	46 [25.8, 61.7]
Live Cells Lost between n1 and n2 (%)	32.7 [16.4, 49.7]
Live Cells Lost between n2 and n3 (%)	13.3 [−10.6, 33.9]
Live Cells Lost between n1 and n3 (%)	40.7 [25.1, 62]

Particularly significant is the cell loss between n1 and n3, which represents the overall cell loss up to the point of flow cytometric measurement. We conclude that the majority of cell loss occurred during centrifugation. However, we are unable to determine with certainty whether this loss affects all cell populations equally or whether specific populations are disproportionately impacted.

### 3.5 Correlation between cellularity and MSC count and percentage in bone marrow concentrate

To evaluate the suitability of using cell count per milliliter of the original Bone Marrow Concentrate (BMC) as an estimate for the number of Mesenchymal Stromal Cells (MSCs), the cell count per milliliter was correlated with the measured number of MSCs. Additionally, to determine whether the cell count per milliliter (referred to as cellularity) is an indicator of a higher percentage of MSCs, the cell count per milliliter was correlated with the percentage of MSCs. A strong correlation between the cellularity of the BMC and the number of MSCs was observed (see Fig. 3A, C, E). Additionally, the potential of cellularity to serve as an indicator for the percentage of MSCs was investigated. For this purpose, the cell count per milliliter was correlated with the percentage of MSCs. No correlation was found, indicating that the cellularity of the sample does not provide information about the percentage of MSCs (see Fig. 3B, D, F).

## 4 Discussion

We collected bone marrow for research purposes from patients undergoing endoprosthesis implantation. The bone marrow was prepared by density gradient centrifugation using the Regenlab BMC system, which can be used for clinical applications such as arthrosis therapy. We evaluated the cellularity and the vitality of the cells using a NucleoCounter. To verify the validity of this measurement of the quality of the BMC, we determined the proportion of MSCs by positive selection of CD271^+^ cells and subsequent flow cytometry, further distinguishing between CD271^+^ CD45^+^ and CD45^−^ cells. A total of 41 samples were collected from a relatively homogeneous patient group.

In our study, the median percentage of CD271^+^ CD45- cells within the bone marrow samples was 0.014, with an interquartile range (IQR) of [0.004, 0.036] These results are consistent with the ranges reported in existing literature (Cuthbert et al., 2012; Müller et al., 2019). El-Jawhari et al. (2020) reported slightly higher cell counts using a vertical centrifugation system, achieving between 1605 and 140,000 cells per ml, with a median of 4470 cells/ml for BMAC samples. This suggests that the vertical centrifugation system may offer advantages in terms of cell yield compared to density gradient centrifugation methods. Such improvements in yield may enhance the quality and quantity of cells available for therapeutic use (El-Jawhari et al., 2020). In another study, Cuthbert et al. (2012) reported a CD271^+^ cell population ranging between 0.001 % and 0.1 %, with a median of 0.026 %, which closely aligns with our findings. This consistency reinforces the reliability of CD271^+^ as a marker for MSCs across different studies and methodologies (Cuthbert et al., 2012).

We observed significant variability in cellularity across samples, with a median of 3.82e+06 cells/ml, with an interquartile range (IQR) of 2.45e+06 to 6.1e+06. Such variation in Bone Marrow Aspirate (BMA) cellularity has been well-documented in the literature, although direct comparisons are difficult due to different methodologies for producing Bone Marrow Concentrates (BMC) and varying aspiration techniques. For example, Pabinger et al. (2021) reported similar cell counts, focusing on CD34^+^ cells, which are more prevalent than CD271^+^ cells in bone marrow (Pabinger et al., 2021).

We also analyzed the bone marrow aspirates collected from different anatomical sites, including the distal femur, proximal femur, and proximal tibia. The comparison revealed notable differences in cellularity and the concentration of CD271^+^ cells across these sites. Specifically, samples collected from the proximal femur exhibited higher concentrations of total nucleated cells and CD271^+^ CD45- cells compared to those from the distal femur and proximal tibia. This suggests that the proximal femur may serve as a more optimal site for harvesting bone marrow with a higher yield of MSCs, potentially improving the effectiveness of BMC preparations for clinical applications. The observed variations emphasize the importance of the aspiration site in influencing the quality and cellular composition of BMCs, which could have significant implications for optimizing regenerative therapies.

Pabinger et al. found no significant difference regarding the BMA cell count between anterior and posterior harvesting of the iliac crest (Pabinger et al., 2021). Overall, there is little literature comparing harvesting sites. However, our results suggest that the selection of the harvesting site could be relevant for clinical applications. In addition to cell quantity and quality, clinical considerations must include choosing a site with low harvesting morbidity. Traditionally, the iliac crest has been preferred due to its low morbidity, and the proximal tibia is also very accessible. If future studies demonstrate a significant superiority of the proximal femur, it may be worth considering harvesting from this site, even if it involves higher morbidity, such as requiring a larger incision to access the bone securely.

A critical observation from our study is the strong correlation between cellularity (total number of cells per ml) and MSC concentration (number of MSCs per ml). This finding is highly relevant for clinical practice it suggests that by measuring the overall cell count in BMCs, a reliable estimate of the concentration of MSCs can be obtained. This would streamline future protocols, as a direct MSC count would no longer be required. Instead, total cellularity could serve as a proxy to determine whether sufficient MSCs are present in the BMC for therapeutic purposes.

We also explored whether the cellularity of the sample could predict the percentage of MSCs. The rationale was that BMC samples with higher total cell counts might be less diluted and therefore contain a higher proportion of MSCs. However, our results did not support this hypothesis. Despite the variability in total cell counts, we found no correlation between cellularity and the percentage of MSCs within the sample. This suggests that higher cellularity does not necessarily indicate a less diluted sample or a higher relative abundance of MSCs. Instead, it appears that even in samples with a high total cell count, the proportion of MSCs remains independent of cellularity.

Variation in Bone Marrow Aspirate (BMA) cellularity has been previously described, although comparability is limited due to differing methods for creating Bone Marrow Concentrates (BMC) and variations in aspiration techniques. For example, Cuthbert et al. (2012) described a significant 7-fold decrease in MSCs after prolonged withdrawal (Cuthbert et al., 2012), while a recent study comparing cell counts and CD34^+^ progenitor cells in BMA using the “Reorientation Technique” showed significantly higher leukocytes and CD34^+^ progenitor cells with this technique (Muthu et al., 2023). We employed a similar technique to minimize dilution effects. Despite the variability in cellularity, we found that cellularity strongly correlated with MSC concentration, demonstrating that adequate stem cell counts can be achieved even at lower cellularity with appropriate collection volumes.

Beyond MSCs, alternative regenerative approaches such as the MSC secretome, extracellular vesicles (EVs), and platelet-rich plasma (PRP) have gained interest due to their potential therapeutic benefits. The MSC secretome, which includes bioactive molecules like cytokines, growth factors, and EVs, offers a cell-free alternative that may reduce risks such as immune rejection and tumorigenesis, while retaining significant regenerative potential (Damania et al., 2018; Redondo-Castro et al., 2017; Hyvärinen et al., 2018; Kearney et al., 2022), Recent studies have demonstrated that MSC-derived exosomes can enhance angiogenesis, reduce inflammation, and modulate the immune response, facilitating recovery in various injury models (Kearney et al., 2022; Mendes-Pinheiro et al., 2019). Similarly, PRP has been shown to enhance tissue repair through its growth factor content, promoting cell proliferation, migration, and differentiation (Khatab et al., 2018; Lee et al., 2017). Our findings on MSC concentration provide a basis for future studies to compare the effectiveness of MSCs, MSC secretome, EVs, and PRP in clinical applications. Combining these approaches may provide synergistic effects, improving outcomes in regenerative medicine, particularly in cases where cell-based therapies face limitations due to immune or ethical challenges.

Significant cell loss was observed during the preparation of Bone Marrow Concentrates (BMC), as detailed in Table 4. The total cell loss between n1 and n2 had a median value of 37.5 %, while the losses between n2 and n3 were much lower, with a median of 16.2 %. A similar pattern was observed for viable cells, with a median loss of 32.7 % between n1 and n2, compared to 13.3 % between n2 and n3. These results indicate that the major cell loss occurred during the centrifugation step, whereas the losses during the CD271^+^ cell isolation process were comparatively minor. This suggests that the centrifugation step was primarily responsible for the reduction in cell numbers, while the magnetic selection columns contributed minimally. Cuthbert et al. (2012) avoided centrifugation and resuspension of the cell suspension to prevent cell loss in the centrifuged supernatant, noting that these steps could lead to inaccuracies in cell frequency measurements. They used the LSR II flow cytometer (BD Pharmingen) and were able to set the event rate to 2000 events per second, which allowed them to avoid additional cell handling steps (Cuthbert et al., 2012). In contrast, our study employed the Guava easyCyte™ 8HT flow cytometer, with a maximum event rate of approximately 100 events per second. Due to this limitation, we had to include an additional positive selection step using magnetic cell separation to reliably detect the rare CD271^+^ cell population.

This limitation underscores the need for methods that do not require such extensive sample preparation for clinical applications. Long-term, we aim to establish a method using NucleoCounter measurements, which we also employed in this study directly on the BMC, to measure cell counts before BMC application. This will allow us to estimate the MSC proportion based on the established baseline. As the MSC percentages in our study align with other published results, we conclude that cell loss during preparation may not significantly impact clinical outcomes.

## 5 Conclusion and future perspectives

This study demonstrated the feasibility of preparing and evaluating BMC using a novel concentration device in elderly patients undergoing elective arthroplasty. The CD271^+^ CD45^−^ cell detection was reliable, and while significant cell loss occurred primarily during centrifugation, we assume this loss has minor relevance to our study, as our results are comparable to values reported in the literature. Future research should focus on identifying variables that could influence cellularity and the percentages of MSCs in bone marrow aspirates. Investigating other cell markers that indicate progenitor populations will also be essential to further refine and improve the quality of BMCs. These insights will enhance protocols to ensure optimal therapeutic outcomes in regenerative medicine. Overall, our results lay the foundation for the further development and establishment of BMC in clinical applications, particularly for treating osteoarthritis and other degenerative conditions. Our analysis of different aspiration sites revealed that the proximal femur may provide a higher yield of MSCs compared to other sites such as the distal femur and proximal tibia. Selecting the optimal harvesting site is crucial to maximize BMC effectiveness while considering factors like morbidity. The proximal femur showed promise for higher MSC yield, but harvesting morbidity must be weighed against benefits. Standardized protocols are needed to balance yield, safety, and procedural ease for clinical use.

## CRediT authorship contribution statement

**Klaus Werner Labarre:** Writing – review & editing, Writing – original draft, Visualization, Validation, Supervision, Software, Resources, Project administration, Methodology, Investigation, Formal analysis, Data curation, Conceptualization. **Peter Ansgar Grathwol:** Writing – review & editing, Project administration, Formal analysis, Conceptualization. **Gerald Zimmermann:** Writing – review & editing, Supervision, Funding acquisition, Conceptualization.

## Funding statement

This study was funded by grants provided to the Department of Trauma Surgery at Theresienkrankenhaus by the Karl Kärcher Foundation for research purposes. No additional funding was received.

## Declaration of competing interest

The authors declare that they have no known competing financial interests or personal relationships that could have appeared to influence the work reported in this paper.

## Data Availability

Data will be made available on request.
